# ReSort enhances reference-based cell type deconvolution for spatial transcriptomics through regional information integration

**DOI:** 10.1093/bioadv/vbaf091

**Published:** 2025-05-27

**Authors:** Linhua Wang, Ling Wu, Guantong Qi, Chaozhong Liu, Wanli Wang, Xiang H -F Zhang, Zhandong Liu

**Affiliations:** Graduate School of Biomedical Sciences, Program in Quantitative and Computational Biosciences, Baylor College of Medicine, Houston, TX 77030, United States; Lester and Sue Smith Breast Center, Baylor College of Medicine, Houston, TX 77030, United States; Graduate School of Biomedical Sciences, Program in Genetics and Genomics, Baylor College of Medicine, Houston, TX 77030, United States; Graduate School of Biomedical Sciences, Program in Quantitative and Computational Biosciences, Baylor College of Medicine, Houston, TX 77030, United States; Graduate School of Biomedical Sciences, Program in Quantitative and Computational Biosciences, Baylor College of Medicine, Houston, TX 77030, United States; Lester and Sue Smith Breast Center, Baylor College of Medicine, Houston, TX 77030, United States; Department of Molecular and Cellular Biology, Baylor College of Medicine, Houston, TX 77030, United States; McNair Medical Institute, Baylor College of Medicine, Houston, TX 77030, United States; Jan and Dan Duncan Neurological Research Institute at Texas Children’s Hospital, Houston, TX 77030, United States; Department of Pediatrics, Baylor College of Medicine, Houston, TX 77030, United States

## Abstract

**Motivation:**

Spatial transcriptomics (ST) captures positional gene expression within tissues but lacks single-cell resolution. Reference-based cell type deconvolution methods were developed to understand cell type distributions for ST. However, batch/platform discrepancies between references and ST impact their accuracy.

**Results:**

We present Region-based Cell Sorting (ReSort), which utilizes ST's region-level data to lessen reliance on reference data and alleviate these technical issues. In simulation studies, ReSort enhances reference-based deconvolution methods. Applying ReSort to a mouse breast cancer model highlights macrophages M0 and M2 enrichment in the epithelial clone, revealing insights into epithelial-mesenchymal transition and immune infiltration.

**Availability and implementation:**

Source codes for ReSort are publicly available at (https://github.com/LiuzLab/RESORT), implemented in Python.

## 1 Introduction

Sequencing-based spatial transcriptomics (ST) techniques have been groundbreaking in dissecting cell-cell communications within tissues by profiling positional gene expression (Ståhl *et al.* 2016, Berglund *et al.* 2018, Thrane *et al.* 2018, Chen *et al.* 2020, He *et al.* 2020). However, analyzing these datasets remains challenging due to the multi-cell resolution of the sequenced spatial units (referred to as spots). To reveal the spatial organization of cell types and their changes with respect to other biological factors, such as pathology, it is critical to dissect cell type composition at every spot.

In addition to traditional bulk RNA-sequencing deconvolution techniques, such as CIBERSORTx (Steen *et al.* 2020) and MuSiC (Wang *et al.* 2019), many algorithms have been created to deconvolute cell type compositions for ST data. Stereoscope (Andersson *et al.* 2020) uses a negative binomial model to infer cell type proportions from single-cell RNA-sequencing data. SPOTlight (Elosua-Bayes *et al.* 2021) employs non-negative matrix factorization and least squares to estimate proportions. Another method, SpatialDWLS (Dong and Yuan 2021) assesses cell type enrichment and applies weighted least squares for estimation.

However, all the aforementioned methods rely heavily on reference data from either single-cell RNA sequencing or a signature gene expression matrix, from which they learn cell type-specific gene expression to estimate the ratios of different cell types at every location in ST data. Consequently, deconvolution becomes challenging if a proper single-cell reference is unavailable. Moreover, technical noise such as batch and platform effects will affect the accuracy of the deconvolution results, especially when the references are from other experiments (Fig. S1).

Some approaches model platform-specific parameters to adjust for these technical noises. For example, RCTD (Cable *et al.* 2022) and cell2location (Kleshchevnikov *et al.* 2022) estimate gene-based platform effects in Bayesian models. Other methods, such as BayesSpace (Zhao *et al.* 2021), first detects spot-level clusters and uses spot-level information to estimate sub-spot compositions. It circumvents the platform effects within external references by generating a pseudo-internal reference. However, some spot-level clusters from BayesSpace might contain spots with mixed cell types, introducing noises for subspot-level estimations.

To provide a solution from a novel perspective, we propose a Region-based cell type Sorting (ReSort) strategy that creates a pseudo-internal reference by extracting spatial regions from the ST data and leaves out spots that are likely to be mixtures. By detecting these regions with diverse molecular profiles, we can approximate the pseudo-internal reference to accurately estimate the composition at each spot for any sequencing-based ST datasets, bypassing an external reference that could introduce technical noise.

In this study, we simulated ST datasets under different scenarios to demonstrate the efficacy of ReSort, finding that it generally improved the reference-based deconvolution methods’ accuracy by reducing technical effects. We also applied ReSort to a breast cancer ST sample to demonstrate its utility in investigating tumor microenvironments and tumor-infiltrating immune cells.

## 2 Methods

### 2.1 Simulating patterns of region-level cell types

**Figure 1.**
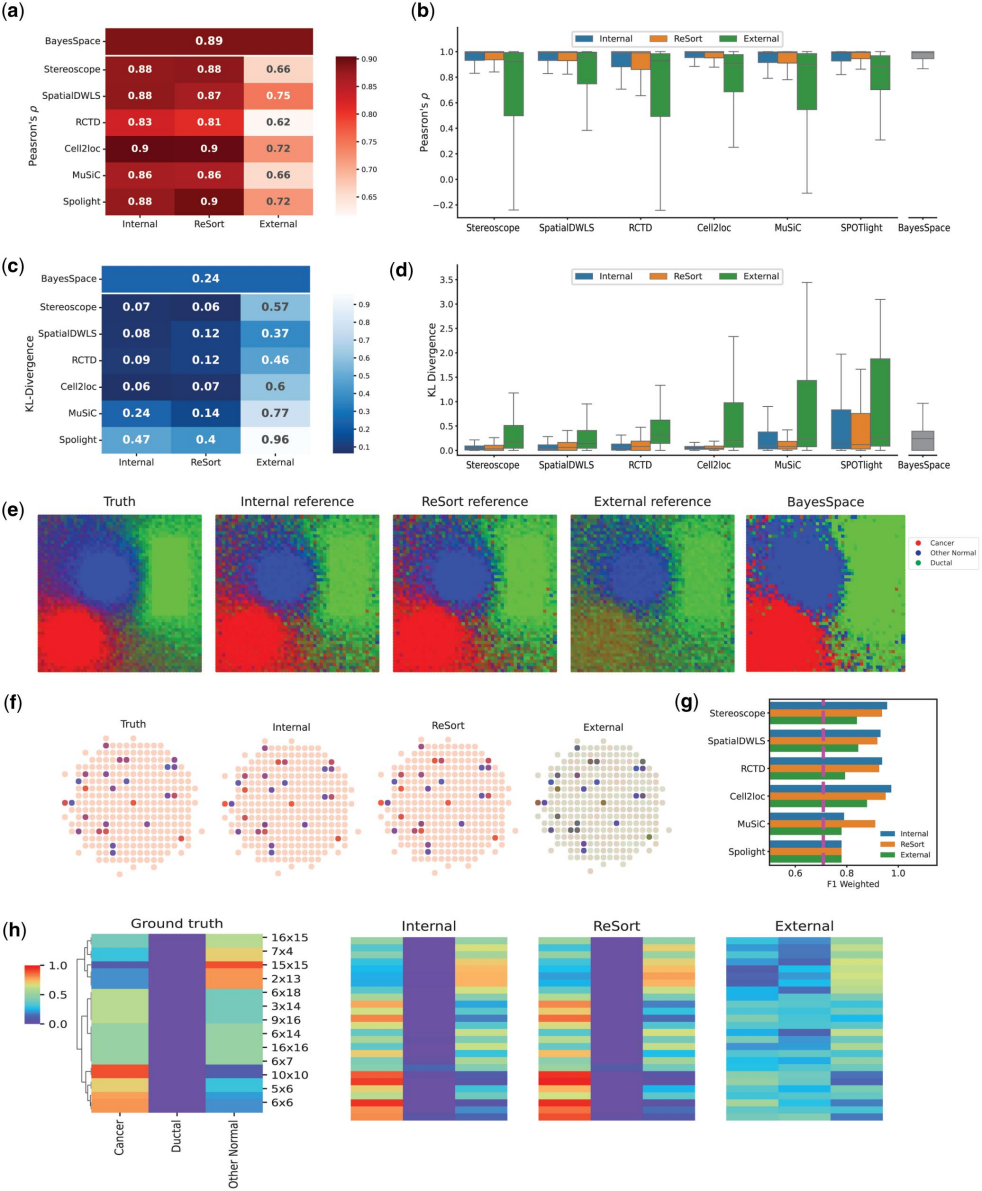
Benchmarking region-level cell type deconvolution performances using simulated ST data. (a–d) Performance comparison evaluated by Pearson’s correlation coefficient (a, b) and KL divergence (c, d). Heatmaps (a, c) show average metrics across all spots with rows as deconvolution methods and columns as the reference for each technique. Box plots (b, d) show the distribution of performances across the spots with columns as deconvolution methods. Box plots are defined with center line (median), box limits (upper and lower quartiles) and whiskers that extend at most 1.5 times of the interquartile range. (e) Visualization of cell-type proportions as RGB images using different references where red, green, and blue channels indicate the abundances of cancer, ductal and other normal cells, respectively. From left to right are simulated ground truth, Stereoscope estimates using internal reference, ReSort strategy and external reference, and BayesSpace estimates. (f) Visualization of the cell type proportions in the cancer region with immune infiltration us. From left to right: using ground truth, Stereoscope with internal, ReSort, external references, and BayesSpace. Spots that have less than 5% immune are in smaller size and 50% transparency for a better contrast. (g) Weighted F-1 score (*x*-axis) for classifying immune-infiltration and pure cancer spots. The *y*-axis indicates the deconvolution methods, with colors indicating reference types. BayesSpace’s performance provides a baseline depicted by a dashed purple line. (h) Heatmaps of cell type proportions in the cancer region with immune infiltration. Each row is a cancer spot with different level of immune infiltration, and each column is a region-level cell type. From the left to right: ground truth with rows clustered using cell type profiles, with internal, ReSort, and external references. The row and column orders are the same across the panels.

We first generated a tissue grid of 2500 spots with a layout of 50 rows and 50 columns. To mimic the pathological organization of tissues, three major regions with homogeneous cell populations—namely, the cancer, ductal, and other normal regions—were simulated. The cancer and normal regions were circular and the ductal region was rectangular (Fig. 1).

To simulate the ratio of cell type r at every out-of-region spot s, we denoted the affinity $A_{s,r}$ between spot s and region r as the inverse of the Euclidean distance between s and the closest point within the region r, with a random Gaussian noise, $\delta_{r}$  (1). We then normalized the proportions to sum to one (2).


(1) $$A_{s,r}=\frac{1}{{\min.}_{s^{\prime }\in r}\mathrm{Dist}(s,s\prime )\;}+\delta_{r}\;$$

(2) $$\tilde{P_{s,\;r}}=\frac{A_{s,r}}{\sum_{r\prime }A_{s,r\prime }}\;$$

Since every spot in Visium ST data captures about ten cells, to transfer cell type proportions to counts we assigned the count of each cell type *r*, denoted by $C_{s,r}$, by multiplying the ratios obtained in (2) by 10 and taking the integer values (3). The proportion values are then re-normalized and saved for later evaluation (4).


(3) $$C_{s,\;r}=\mathrm{INT}(10*\tilde{P_{s,\;r}})\;$$

(4) $$P_{s,\;r}=\frac{C_{s,r}}{\sum_{r\prime }C_{s,r\prime }}\;$$

### 2.2 Sample cells and reads to form the ST count matrix

We sampled $C_{s,\;r}$ cells from type r without replacement for every spot s. To make the library size of each spot in the simulated ST data comparable to that of actual Visium datasets, we down-sampled 10% of the gene counts from every sampled cell to keep the number of reads in every simulated spot at the same magnitude as the Visium data. All cells' sampled gene counts were then aggregated to make a simulated mini-bulk gene expression count matrix.

### 2.3 Simulate tumor spots with immune infiltration

To simulate cases in which one cell type infiltrated a core region of other cell types—e.g. immune infiltration into the cancer area—we randomly sampled 10% of the spots in the cancer area and added immune cells artificially. The proportion of immune cells for every such spot was sampled from a Gaussian distribution (μ  =  0.5, σ  =  0.2). The resampled count matrix was then generated using the aforementioned steps (1–4).

### 2.4 Extract spatial regions using MIST

We used a previously developed method, MIST (Wang *et al.* 2022), to extract homogeneous regions to detect spatial regions. MIST combines the molecular similarity and the physical adjacency among spots to determine highly homogeneous regions in the sequenced tissue.

MIST normalizes the count matrix by the library size and transforms the resulting gene expression values at a log scale. It then performs principal component analysis on the normalized gene expression values and calculates a similarity score for spatially adjacent spots using the first *k* principal components. The similarity score serves as the weight of the edge *E* =< *u*, *v* > for spot *u* and *v*. MIST, then filters out edges with low weights using an automatically optimized threshold epsilon. Each connected component in the remaining graph, with more than *m* nodes, serve as the molecular regions, where *m* is a constant with the default value of 40 for Visium data.

To validate the accuracy of the annotations on our simulated sample, we calculated a Rand Index of 0.74 between annotated regions and the ground truth using the Python scikit-learn package. Figure S2b shows the visual concordance between MIST-detected regions and the ground truth.

### 2.5 Region-level cell type deconvolution

In the simulation study, we grouped the fourteen cell types in the PDAC-A and PDAC-B single-cell RNA-sequencing data into three region-level cell types: cancer, ductal, and other normal cells.

For ReSort, this task does not require additional single-cell RNA-sequencing references and avoids technical effects when estimation is based on external references. A curated reference matrix *Ω* was generated. Every row in *Ω* is a spot with an annotated region-level cell type, and every column is a gene. *Ω* contains sufficient source information required by all the compared deconvolution methods. For every spot, we denote $\tilde{Y_{T}}$ as the estimated proportion for region-level cell type *T*, with all proportions summed to be 1.

### 2.6 Finer cell type deconvolution

We define finer cell type deconvolution as the task of estimating the proportions of finer cell types, such as macrophages. These finer cell types generally do not form topological structures in the ST samples, so they could not be directly extracted using MIST.

To achieve this goal, ReSort used a two-step deconvolution strategy. It first applied the region-level deconvolution mentioned above to estimate the proportions of every region-level cell type *T*’s proportion, denoted as $\tilde{Y_{T}}$. It then used an external reference *ω*—either single-cell RNA-sequencing data or a signature matrix, such as immune signature matrix LM22 (Chen *et al.* 2018, Steen *et al.* 2020)—to estimate the finer cell types’ proportions, $\tilde{Y_{T}}$, for each sub-type t. The final estimation of the proportion of subtype t is then calculated in (5).


(5) $$Y_{t}=\;\tilde{Y_{T}}*\tilde{Y_{t}}\;$$

## 3 Results

Here, we introduce the ReSort strategy to address technical noises, such as batch and platform effects (Fig. S1), in reference-based cell type deconvolution algorithms. ReSort first extracts spatial regions from the ST sample using our published method, MIST (Wang *et al.* 2022). Spots within each region have similar molecular profiles to each other and are likely to be dominated by one region-level cell type, such as cancer. The advancement of ST, such asVisium, provides high-resolution spatial profiles with thousands of spots, allowing ReSort to reliably leverage the detected regions, each of which contains considerable number of spots, to form a pseudo-internal reference.

ReSort enhances the decomposition of finer cell types, such as macrophages, that will not form a spatial region in ST samples. Specifically, ReSort applies a two-step approach: it first deconvolutes the regional composition at each spot, then uses the regional compositions as priors to adjust the estimations from external-reference-based cell type deconvolution methods (see Methods). By doing so, ReSort constrains estimations of finer cell types by their regional (e.g. tumor or non-tumor) proportion and thus reduces errors.

### 3.1 ReSort accurately estimated region-level cell type compositions

*In silico* simulation has been widely used to assess the efficacy of computational approaches by simulating faithful ground truth (Jin and Liu 2021). Here, we simulated ST data with 2500 pseudo-spots on 50 by 50 grids (Fig. 1c, Fig. S2a) using a public pancreatic ductal adenocarcinomas (PDAC) single-cell RNA-sequencing data from one patient (Moncada *et al.* 2020). This dataset was referred to as the internal reference since it is an ideal reference for deconvolution. However, such a reference does not exist in real deconvolution tasks. Thus, an external reference is often used by reference-based deconvolution methods. The single-cell RNA-sequencing data from another PDAC patient was introduced as an external reference to simulate actual deconvolution scenarios.

We first benchmarked the accuracy of six state-of-the-art deconvolution methods, including stereoscope (Andersson *et al.* 2020), spatialDWLS (Dong and Yuan 2021), RCTD (Cable *et al.* 2020), cell2location (Kleshchevnikov *et al.* 2022), MuSiC (Wang *et al.* 2019), and SPOTlight (Elosua-Bayes *et al.* 2021), on three region-level cell types—cancer, ductal, and other normal cells—with the external reference, internal reference, and the ReSort strategy. All reference-based approaches were further compared with a reference-free approach, BayesSpace.

To quantify the linear alignment between the estimated and ground truth cell type proportions in each spot, we calculated Pearson’s correlation coefficient, *ρ* (Fig. 1a and b). The coefficient ranges from −1 to 1, with 1 indicating perfect correlation, 0 no correlation, and negative values indicating negative correlation. We found that ReSort significantly increased *ρ* by 0.18 on average compared to the external reference (*P* < 1e−10, two-sided paired t-test, *N* = 2500). The difference in *ρ* compared to the internal reference was negligible (Δρ = −0.002).

To assess spot-level distributional divergence between the estimated and ground truth cell type proportions, we calculated relative entropy or Kullback–Leibler (KL) divergence (Fig.1c and d). KL divergence ranges from 0 to positive infinity, with 0 indicating perfect alignment and higher values indicating greater disparity. On average, ReSort achieved three times lower KL divergence than the external reference (*P* < 1e−10, two-sided paired t-test, *N* = 2500) and 42% lower KL divergence than the internal reference (*P* < 1e−10, two-sided paired t-test, *N* = 2500). Compared to BayesSpace, ReSort achieved much lower KL divergence in five out of six cases.

To compare the estimated spatial patterns using different references, we transformed the proportions of cells into red-, green-, and blue- (RGB) colored channels where the strength of red, green and blue indicates the abundance of tumor, ductal, and other normal cells, respectively (Fig. 1e, Fig. S2b). ReSort showed a similar RGB image to those of ground truth and the internal reference (Fig. 1e). In contrast, the external reference created an inaccurate pattern, especially in the tumor region. This result may have been caused by the heterogeneity in ductal and tumor profiles between the internal and external references (Fig. S1), highlighting the challenges of using an external reference when deconvoluting ST data.

When comparing different reference-based approaches, we observed that although all methods achieved a similar level of Pearson’s correlation coefficients, stereoscope and cell2loc had much lower divergence than other methods when using either an internal reference or ReSort. When using an external reference, spatialDWLS achieved the highest Pearson’s correlation coefficient (0.75) and lowest KL divergence (0.37) (Fig. 1). Notably, our findings were concordant with another benchmarking study (Li *et al.* 2022), which showed that cell2location, spatialDWLS, RCTD, and stereoscope significantly outperformed SPOTlight.

### 3.2 ReSort identified immune infiltration in a simulation study

ST samples might pose greater challenges than the scenario described above. For example, tumor samples may be infiltrated by immune cells that are enclosed in the tumor. To simulate such cases, we randomly added immune cells to 10% of the tumor spots to mimic immune infiltration (Fig. 1f, left most). We then ran all the deconvolution methods previously mentioned with the three references: internal, external, and ReSort, respectively.

When using Stereoscope as the underlying reference-based deconvolution approach, we observed that ReSort accurately revealed immune infiltrating spots within the cancer region (Fig. 1f and h). To quantify various approaches’ abilities in detecting immune infiltrating events, we assessed their performances in classifying two classes of tumor spots: immune-infiltrating tumor spots and pure tumor spots. Due to the imbalanced number of spots in each class, we evaluated precision, recall, and F1 score for each class. We also calculated a weighted F1 score to combine the F1 scores from both classes with weights defined by their relative sizes. Figure 1g shows that ReSort achieves weighted F1 scores similar to the internal reference and outperforms the external references and the result from BayesSpace, which is another reference-free region-level cell type deconvolution approach. Interestingly, spatialDWLS, stereoscope, and RCTD showed much higher F1 scores than MuSiC and SPOTlight.

Additionally, the external reference failed all deconvolution methods in classifying pure tumor spots, resulting in lower F1 scores in the pure cancer class (Fig. 1g, Fig. S8). These results further highlight that batch effects impaired these methods’ ability to distinguish between ductal and cancer cell types (Fig. S1). It also suggests ReSort’s utility in reducing the technical variation that may have existed in the external reference. In addition, MuSiC tends to predict more false immune-infiltrating spots, resulting in a lower recall score when classifying pure tumor spots and lower precision score when classifying immune-infiltrated spots. However, ReSort helped reduce MuSiC’s errors with a higher weighted F1 of 0.91 compared to 0.78 using the internal reference (Fig. 1a–d).

### 3.3. ReSort accurately estimated compositions of finer cell types

Accurately deciphering the composition of ST samples based on their region-level cell types yields crucial insights into regional composition and pathological characteristics. However, it is equally essential, particularly in cancer studies, to profile specific cell types like immune subtypes within pathological tissue samples. Yet, profiling these cell types without a reference presents challenges since not all cell types exhibit distinct spatial clusters in ST data. To effectively address this challenge, ReSort endeavors to enhance reference-based deconvolution tools by curbing the errors that cascade from the region level. This strategic refinement aims to improve the precision of the process. For each spot in the dataset, ReSort initiates the process by estimating regional compositions or proportions attributed to region-level cell types. Subsequently, it establishes constraint priors for the finer secondary cell types linked to each region-level cell type. These constraints play a crucial role in determining the adjusted cell type abundances, specifically for external reference-based deconvolution methods (METHOD: Equation 5). In this way, ReSort brings forth a more accurate and nuanced approach to profiling cell types within ST data.

We used the same simulated immune-infiltrated PDAC sample to evaluate ReSort’s performance in the finer cell type deconvolution. Here, we excluded BayesSpace in benchmarking experiments due to its inability to estimate proportions of finer cell types.

**Figure 2.**
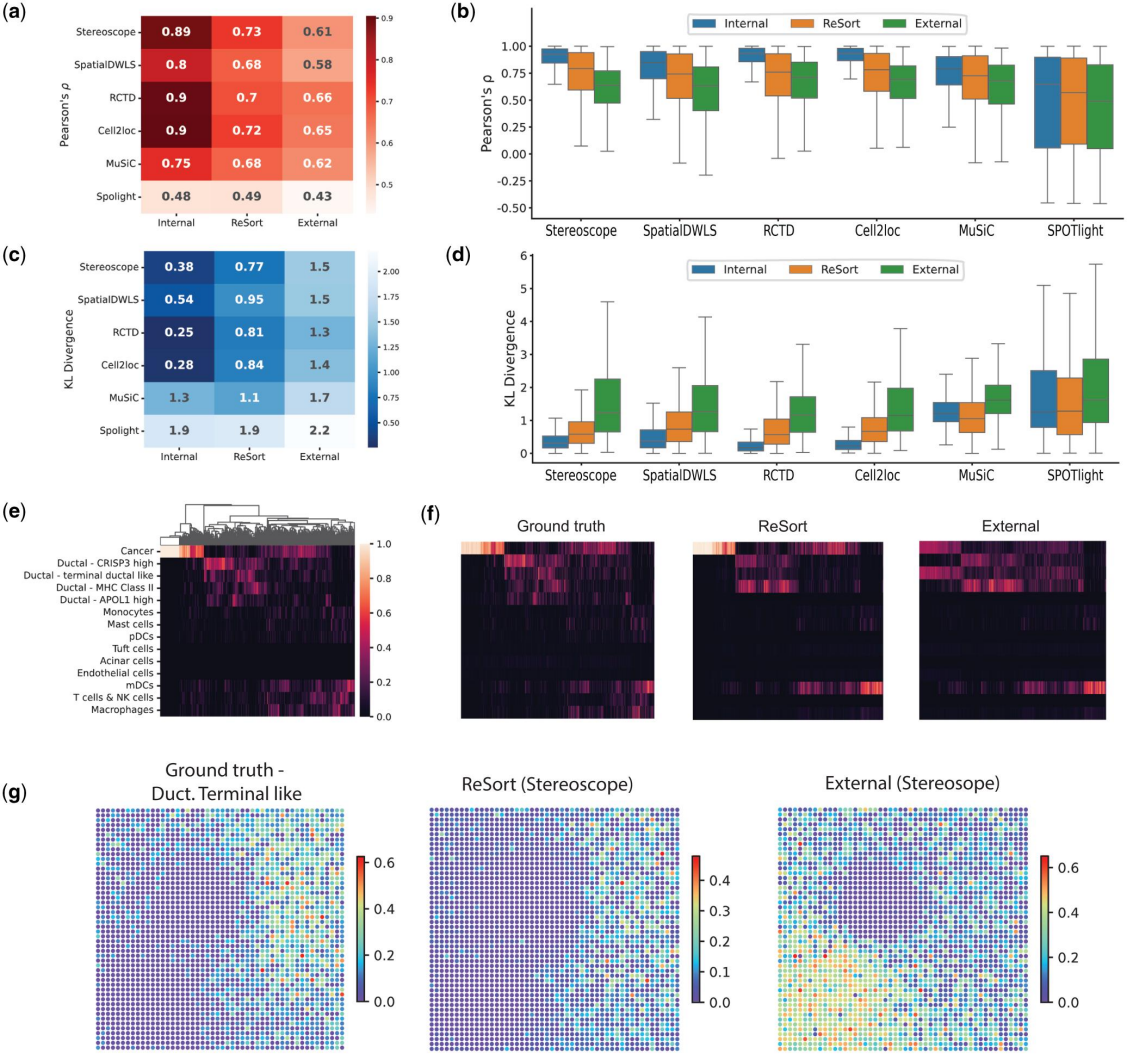
Benchmarking finer cell type deconvolution performances using simulated ST data. (a–d) Performance comparison evaluated by Pearson’s correlation coefficient (a, b) and KL divergence (c, d). Heatmaps (a, c) show average metrics across all spots with rows as deconvolution methods and columns as the reference for each technique. Box plots (b, d) show the distribution of performances across the spots with columns as deconvolution methods. Box plots are defined with center line (median), box limits (upper and lower quartiles) and whiskers that extend at most 1.5 times of the interquartile range. (e) Ground truth heatmap of cell type proportions (rows) across all the spots (columns). The spots are clustered. (f) Heatmaps of Stereoscope estimated cell type proportions. From the left to right: Sterescope with internal, ReSort, and external references. The row and column orders are the same with panel (e). (g) Spatial patterns of the cell type Ductal -Terminal like using ground truth, Stereoscope with ReSort and external reference (left–right).

When using ReSort as compared to the external reference, we observed an 18% increase in Pearson’s correlation coefficient (*P* < 1e−10, two-sided paired t-test, *N* = 2500) and 79% decreased KL divergence (*P* < 1e−10, two-sided paired t-test, *N* = 2500) on average (Fig. 2a–d). A-Taken together, ReSort improved all reference-based deconvolution methods’ performances in both metrics.

It is also of great importance to note that when using an¬¬ external reference, RCTD and Cell2location outperformed other deconvolution approaches by yielding higher Pearson’s correlation coefficients and the smaller KL divergence scores (Fig. 2a and c). These results were consistent with the platform-effect-aware design of RCTD and cell2location. Both methods were designed to reduce platform effects by estimating and removing gene-specific platform factors.

When using ReSort, stereoscope achieves the highest Pearson’s correlation coefficient and the lowest KL divergence across all experiments (Fig. 2a and c). This result suggests the importance of carefully selecting references for complex models like stereoscope, a deep learning-based model.

To gain a deeper understanding of how ReSort enhances Stereoscope's performance on an individual cell-type basis, we generated a heatmap illustrating Stereoscope's estimated proportions of cell types and compared them to the ground truth (Fig. 2e). Our analysis revealed a noteworthy distinction: ReSort’s cell-type heatmap aligns closely with both the ground truth and the estimates derived from the internal reference, in stark contrast to the utilization of an external reference (Fig. 2f). Upon delving into a specific cell type, such as terminal-like ductal cells, a subtype of ductal cells, we unveiled a significant disparity. The application of an external reference inaccurately attributed a substantial number of Ductal Terminal-like cells to the cancer region. Conversely, ReSort accurately depicted the presence of Ductal Terminal-like cells within the Ductal region and its neighboring areas (Fig. 2g).

**Figure 3.**
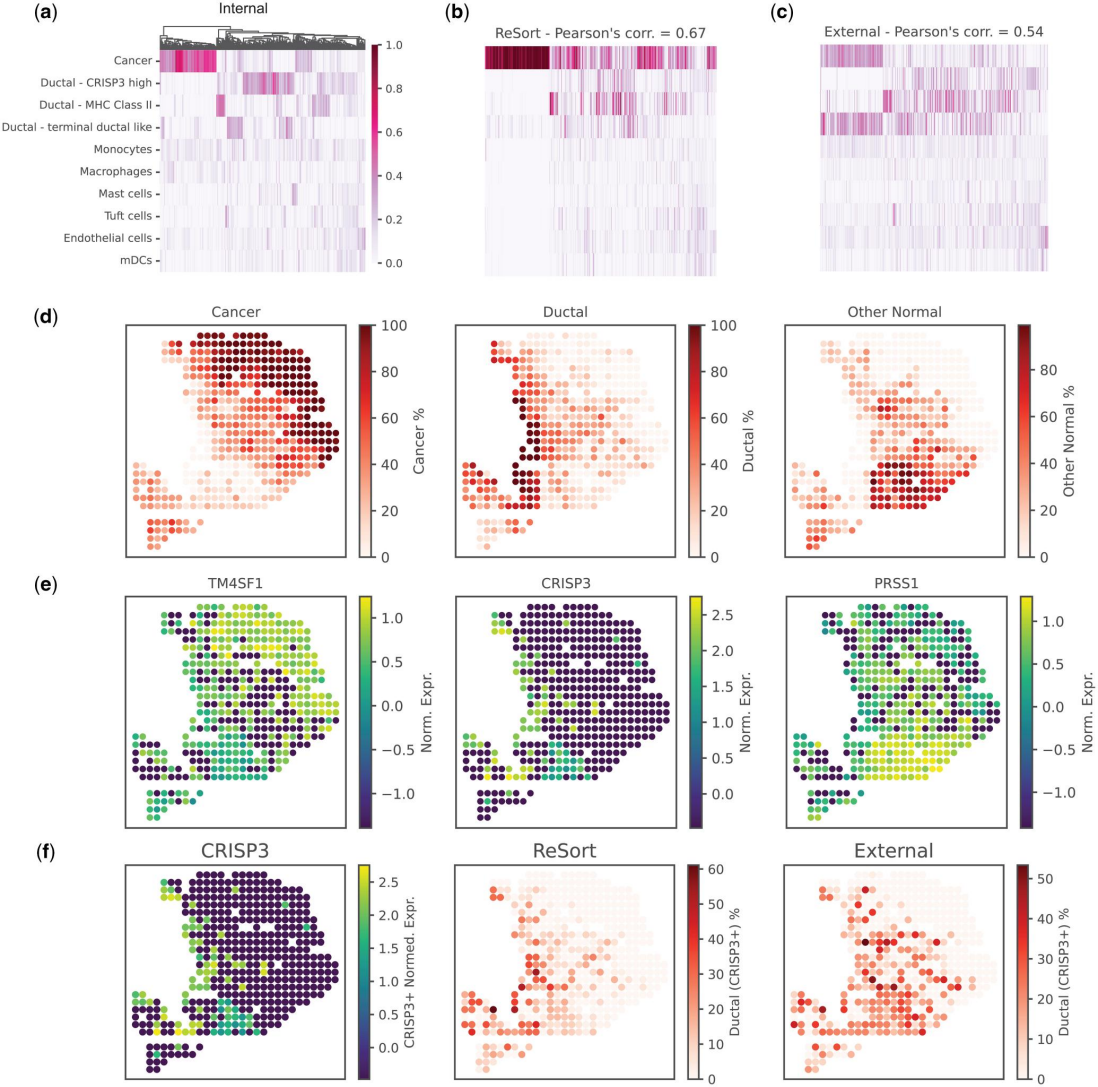
Applying ReSort with stereoscope on a Pancreatic Ductal Adenocarcinoma ST data. (a) Cell types’ heatmap using Stereoscope with single-cell RNA-seq reference data from the same tissue sample (referred to as internal reference). Rows indicate cell types while columns indicate clustered spots. (b, c) Cell types’ heatmap using Stereoscope with ReSort (b) and single-cell RNA-seq reference data from another tissue (referred to as external reference). (d) ReSort estimated region-level cell type proportions. From left to right are Cancer, Ductal and Other Normal. (e) Region-level cell types’ marker genes’ spatial patterns. From left to right are TM4SF1 (Cancer), CRISP3 (Ductal), and PRSS1 (Other Normal). (f) CRISP3’s expression pattern (left), ReSort (middle), and external-reference (right) estimated cell type spatial distributions using Stereoscope.

To showcase the effectiveness of ReSort in real-world ST datasets, we employed Stereoscope with ReSort to reanalyze ST data from pancreatic ductal adenocarcinomas (PDAC) (Moncada *et al.* 2020). Since an established ground truth cell type profile is unavailable, we compared the outcomes generated by ReSort with those derived from the single-cell RNA-seq data of the same sample (referred to as the internal reference, Fig. 3a). Our analysis revealed a significant Pearson's correlation score of 0.67 when utilizing ReSort (Fig. 3b, Fig. S4), a notably higher value compared to the employment of an external reference (Fig. 3c, Pearson's correlation = 0.54). Additionally, we found that ReSort's estimations of spatial patterns for cell types at the region level align closely with the expression patterns of marker genes specific to each of these cell types (Fig. 3d and e). Further investigation into a specific and more detailed cell type, CRISP3+ ductal cells, showcased ReSort's distinct advantage in enhancing cell type estimates compared to the use of an external reference (Fig. 3f).

**Figure 4.**
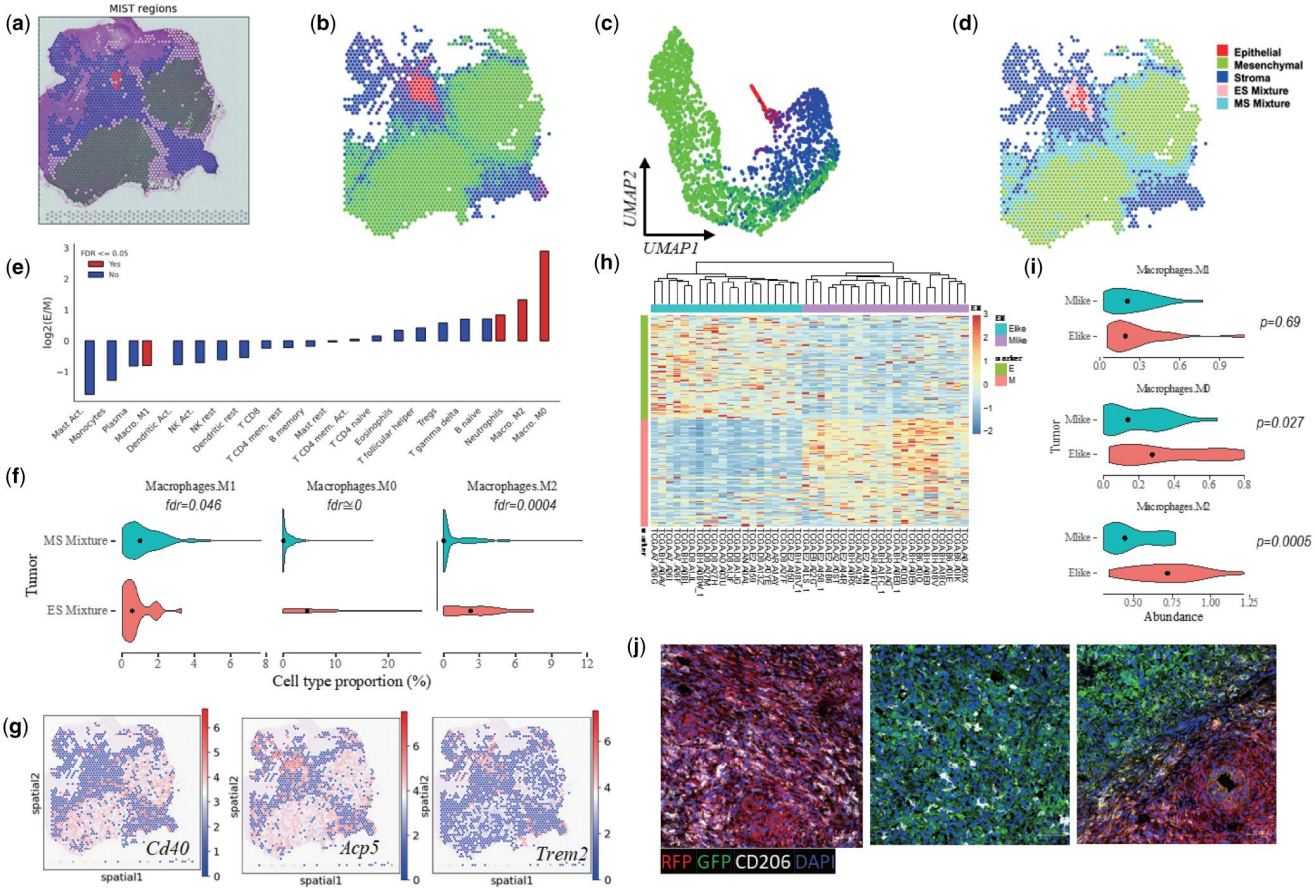
Applying ReSort to a breast cancer tumor with epithelial and mesenchymal tumor clones. (a) Molecular regions detected and mapped to the H&E staining image by MIST.^14^ Red: epithelial clone; green: mesenchymal clone; blue: stroma clone. (b, c) Spatial (b) and UMAP (c) visualization colored by RGB channels using ReSort-estimated cell type proportions. (d) Stratifying spots into a pure epithelial clone (red), mesenchymal clone (green), stroma (blue), epithelial microenvironment (pink), and mesenchymal microenvironment (cyan). (e) Bar plot showing log2 of average fold changes (*y*-axis, *N* = 1 per bar) in LM22 (*x*-axis) immune cell types’ proportions in epithelial versus mesenchymal. (f) Violin plot comparing macrophage subtype’s proportions in epithelial and mesenchymal microenvironments (left to right: M1, M0, M2). Wilcoxon rank sum test used for statistical test with FDR adjusting for multiple comparisons. (g) *In situ* visualization of marker genes’ expression of macrophage subtype M1 (Cd40), M0 (Acp5), and M2 (Trem2). Expression values are colored from blue to red indicating increasing levels. (h) Epithelial-mesenchymal marker genes’ expression in 20 E-like and 22 M-like breast cancer tumors in TCGA. (i) Violin plot comparing macrophage subtype’s proportions in E-like and M-like TCGA tumors (top to down: M1, M0, M2) with mean values marked as black dots. Statistical significances were derived using t-test without multiple-comparison adjustment. (j) Histochemical staining images. RFP, epithelial; GFP, mesenchymal; CD206, M2; DAPI, cell nucleus.

### 3.4 ReSort discovered immune differences in the epithelial and mesenchymal clones of mouse breast cancer tumors

Epithelial-to-mesenchymal transition (EMT) is an important biological process during which cells lose polarity and show decreased adhesion and increased mobility (Kalluri and Weinberg 2010). In cancer development, tumor cells at the primary site undergo EMT to gain higher migratory and invasiveness to metastasize to secondary organs, leading to a worse prognosis in clinical studies (Taube *et al.* 2010, Mak *et al.* 2016, George *et al.* 2017). Studies have shown that various infiltrating immune cells shape the tumor ecosystem at different EMT states, thus affecting treatment outcomes (Polyak *et al.* 2009, Gentles *et al.* 2015, Derynck and Weinberg 2019). How tumors with different EMT states co-evolve with the microenvironment yet retain a relatively distinct ecosystem is very important to cancer treatment but is less well understood.

To better understand the process of EMT in breast cancer, we applied ReSort to a mouse breast tumor sample containing both epithelial and mesenchymal tumor clones. ReSort first detected three regions in the sample using the MIST algorithm (Wang *et al.* 2022) (see Method), including an epithelial tumor (E) region, a mesenchymal tumor (M) region, and a stroma (S) region (Fig. 4a, Fig. S5). Since each region contained spots with high intra-region transcriptional similarities, using these spots as a pseudo-internal reference allowed for accurate estimation of the proportions of region-level cell types across the sample.

To validate the estimation’s accuracy, we colored the spots on the tissue and in the UMAP using the RGB channels representing the proportions of the corresponding region-level cell types (Fig. 4b and c). Specifically, we used red for the epithelial tumor, blue for stroma, and green for the mesenchymal tumor. While pure E, M, and S spots formed clusters in both the H&E image and the UMAP, smooth transitions of colors were simultaneously observed at the boundary points in both figures (Fig. 4b and c). These results suggest that the ReSort-estimated region-level cell types’ proportions represent both the physical and molecular structures of the data, preparing solid prior probabilities for secondary cell types’ deconvolution.

To estimate the roles of different immune cell types in the EMT process, we further stratified the admixture spots into epithelial-stroma (ES, *N* = 32) and mesenchymal-stroma (MS, *N* = 458) mixture spots (Fig. 4d), which represented the microenvironments of epithelial and mesenchymal tumors. Using a two-step ReSort strategy combined with CIBERSORTx (Steen *et al.* 2020) and LM22 signature matrix, we estimated the proportions of 22 immune cell types in the ES and MS spots and statistically compared their compositions.

We observed that ES had 650% elevated M0 macrophages (*P* = 8 × 10e−34), t-test adjusted by multiple comparisons, 150% enriched M2 (*P* = 4e−4, t-test adjusted by multiple comparisons), and 179% increased neutrophils (*P* = .046, t-test adjusted by multiple comparisons). In contrast, MS had 174% elevated M1 macrophages (*P* = .046, t-test adjusted by multiple comparisons) (Fig. 4e and f). Accordingly, we found significantly enriched expression of M0’s marker gene Acp5 (fold change = 150%, *P* = 7e−8, Wilcoxon rank-sum test) and M2’s marker gene Trem2 (fold change = 68%, *P* = 7e−6, Wilcoxon rank-sum test) in the epithelial microenvironment (Fig. 4g). In contrast, Cd40, an M1 marker (Lim *et al.* 2022), was significantly activated within the mesenchymal microenvironment (fold change = 146%, *P* = 5e−4, Wilcoxon rank-sum test) (Fig. 4g).

Using triple-negative breast cancer tumors (TNBC) from The Cancer Genome Atlas (TCGA), we validated that M0 and M2 were more enriched in epithelial-like TNBC tumors. We first extracted epithelial-like (*N* = 22) and mesenchymal-like (*N* = 20) TNBC tumors from TCGA (The Cancer Genome Atlas Network 2012) using marker genes obtained from the ST sample (Fig. 4h). We then used CIBERSORTx (Steen *et al.* 2020) to estimate the abundance of immune cell types in each tumor. We observed significantly enriched M0 (*P* = .03, Wilcoxon rank-sum test) and M2 (*P* = 7e−4, Wilcoxon rank-sum test) in epithelial-like TNBC tumors (Fig. 4i). However, we did not observe significantly enriched M1 in the mesenchymal-like TNBC tumors (*P* = .69, Wilcoxon rank-sum test).

To further validate our findings, we performed histochemical staining of mouse breast cancer tumors with both epithelial and mesenchymal clones. Specifically, we used CD206 to stain M2 cells and CD40 for M1 cells. Due to a lack of M0-specific cell surface marker, we did not stain M0. Both epithelial and mesenchymal clones showed M2 signals when cultured separately (Fig. 4j, left and middle), but we observed a substantially increased amount of M2 signals in the epithelial clone when both clones were cultured together (Fig. 4j, right). M1 staining was not enriched in the mesenchymal clone (Fig. S6).

### 3.5 ReSort facilitates the discovery of tumor-infiltrating immune cells in mesenchymal mouse breast cancer tumors

Advancement of ST techniques enables the profiling of spatial cell type enrichments, which is not possible in either bulk RNA-sequencing or single-cell RNA-sequencing datasets. Our ReSort pipeline, in contrast, was able to take advantage of this spatial information to research an important biomedical topic—ie, tumor infiltrating immune cells, which play a critical role in disease outcomes (Bense *et al.* 2017, Zhou *et al.* 2019).

**Figure 5.**
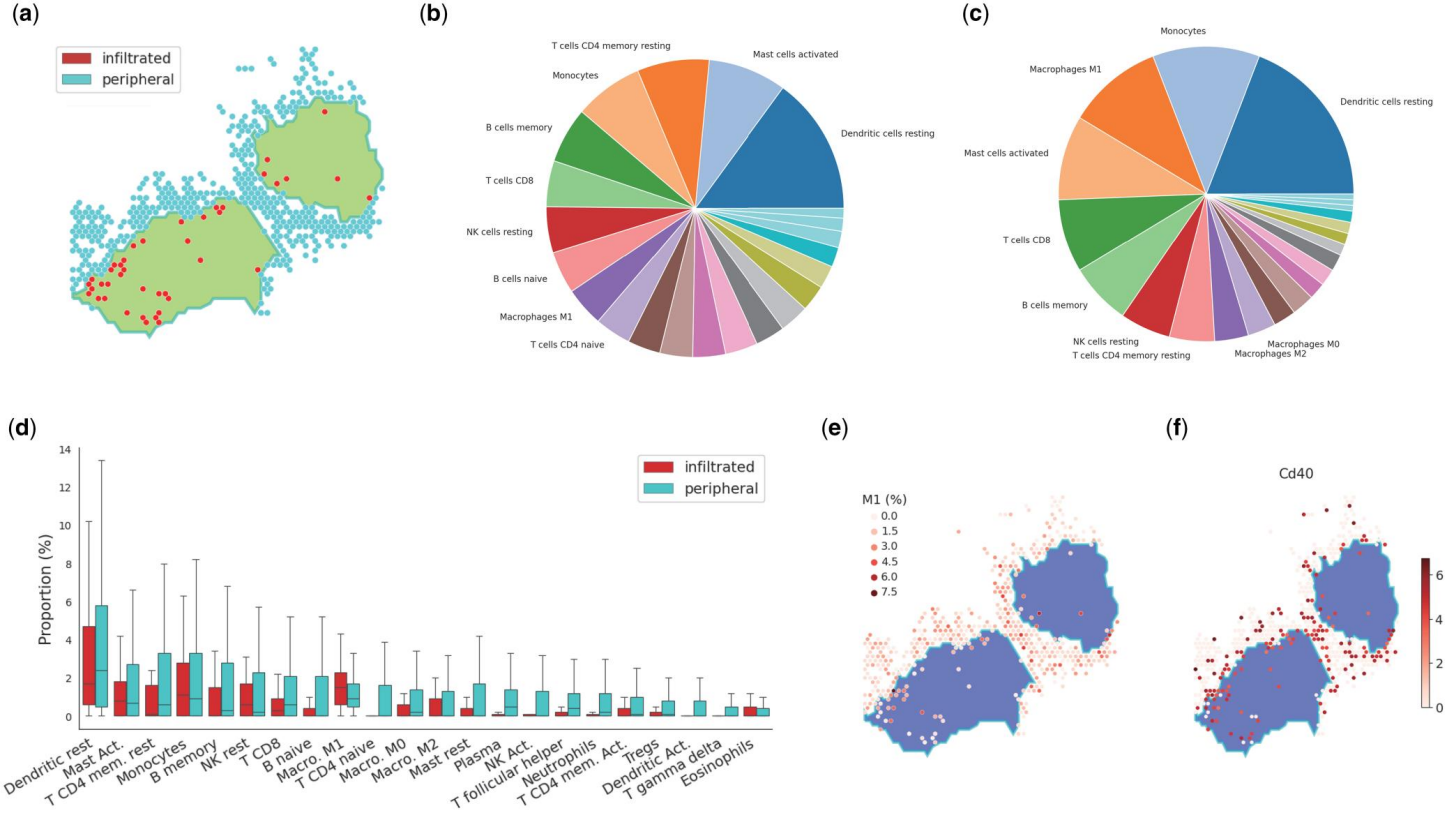
Applying ReSort to investigate immune infiltration in the mesenchymal clone of the breast cancer tumor. (a) Stratifying mesenchymal-stroma mixed spots into peripheral microenvironment (cyan) and immune-infiltrated tumor spots (red) separated by the boundary detected by MIST. (b, c) Pie charts of LM22 immune cell types’ relative proportions in the peripheral (b) and infiltrated (c) immune-tumor mixed spots estimated by ReSort. (d) Box plots of LM22 (*x* axis) immune proportions of cell types (*y* axis) estimated by ReSort. Box plots are defined with center line (median), box limits (upper and lower quartiles), and whiskers that extend at most 1.5 times the interquartile range. (e, f) *In situ* spatial visualization of M1 macrophage’s proportions (e) and its marker gene Cd40’s expression (f) in the mesenchymal microenvironment where the density of redness indicates higher values.

To understand tumor-infiltrating immune cells as compared with peripheral immune cells in the mesenchymal type of mouse breast cancer tumors, we further categorized MS spots as infiltrated or peripheral based on their location in the tissue sample, separated by the mesenchymal tumor region’s boundary as detected by MIST (Wang *et al.* 2022). Infiltrated MS spots are found within the tumor while peripheral MS spots reside in the peripheral area outside the tumor (Fig. 5a).

We then compared the most abundant immune cell types and found that naïve B-cells (rank = 8) and naïve CD4 T cells (rank = 10) ranked within the top 10 of the peripheral tumor microenvironments but were not in the top 10 of the infiltrated spots (Fig. 5b and c). Moreover, the relative abundance rank of M1 macrophages increased more in the infiltrated spots (rank = 3) than in the peripheral spots (rank = 9) (Fig. 5b and c).

Notably, M1 macrophage was the only immune cell type with significantly higher proportions in the infiltrated versus peripheral MS spots (fold change = 42%, *P* = .017, t-test adjusted by multiple comparisons) (Fig. 5d and e). We observed that most infiltrated spots highly expressed M1’s marker gene, Cd4026, while many peripheral spots did not express it at all (Fig. 5f). This result suggests that M1 macrophage is more actively recruited into the tumor center rather than being gated by the boundary, like other immune cell types. Since M1s are anti-tumor macrophages (Lim *et al.* 2022), we believe further investigation of their role in human EMT samples is critical to understanding and facilitating treatment.

## 4 Discussion

In this study, we designed a Region-based digital cell Sorting (ReSort) strategy for sequencing-based ST data sets. ReSort addresses challenges posed by technical noise, such as batch and platform effects, by creating a pseudo-internal reference. This approach enhances the accuracy of reference-based deconvolution methods, particularly when external references introduce inaccuracies. Simulation studies demonstrated that ReSort significantly improves performance metrics, including Pearson’s correlation coefficient and KL divergence, achieving results comparable to those of an ideal internal reference. Additionally, ReSort effectively estimates finer cell type compositions by incorporating regional priors, reducing errors stemming from reference heterogeneity and demonstrating its versatility.

The application of ReSort to mouse breast cancer tumors revealed significant insights into the polarization of macrophages in epithelial and mesenchymal clones. Specifically, the enrichment of M1 macrophages in the mesenchymal tumor microenvironment and M0/M2 macrophages in epithelial clones underscores the importance of understanding immune cell roles in EMT. Additionally, ReSort identified distinct tumor-infiltrating immune cell patterns, with M1 macrophages actively recruited into the tumor center, highlighting their anti-tumor role. These findings align with prior studies suggesting tumor-associated macrophages are critical to immune therapy outcomes. Since many other studies have reported that tumor-infiltrating immune cells and tumor-associated macrophages are critical to the outcome of immune therapies (Bense *et al.* 2017, Tariq *et al.* 2017), we believe further validation using human samples is essential to provide greater insights for treatment.

While we see that ReSort could be reliably applied for region-level cell type deconvolution and improve reference-based deconvolution methods for finer cell type-level deconvolution, it certainly has some limitations. First, while we showed that ReSort could enhance the understanding tumor tissues’ cell compositions, we expect that there will be limited room for ReSort to enhance cell type deconvolution for tissues where complex cell types do not form spatial regions. For example, brain samples where some cell types, e.g. glial cells and neurons, are mixed in all the major regions as shown by Chen *et al.* Second, while ReSort could be used to enhance reference-based deconvolution methods, it cannot address the issue where some cell types are missing in the reference data. We think it is an intrinsic problem for reference-based deconvolution approaches. A potential strategy and future direction to address this issue is to integrate diverse references that could cover a more comprehensive reference spectrum.

## Supplementary Material

vbaf091_Supplementary_Data

## Data Availability

Single-cell RNA-sequencing of pancreatic ductal adenocarcinomas was obtained from Gene Expression Omnibus under accession number GSE111672. Simulated ST and the mouse PyMT-M and PyMT-N Visium ST sample were deposited at Zenodo (doi: 10.5281/zenodo.7434870).
