# Supplementary material for: ReSort enhances reference-based cell type deconvolution for spatial transcriptomics through regional information integration
Source: Bioinform Adv. 2025 May 27;5(1):vbaf091. doi: 10.1093/bioadv/vbaf091 (PMC12161990; doi:10.1093/bioadv/vbaf091)
Supplement: vbaf091_Supplementary_Data [file vbaf091_supplementary_data.docx]

**Supplementary Methods**

***Single-cell RNA-sequencing data collection***

Single-cell RNA-sequencing of pancreatic ductal adenocarcinomas was obtained from Gene Expression Omnibus under accession number GSE111672. We used the filtered raw count matrix of cells from PDAC-A as building blocks to simulate RNA profiles of spatial transcriptomes and referred to it as the internal reference. In contrast, the single-cell RNA-sequencing data from the other patient (PDACA-B) used in the same study^16^ was referred to as the external reference.

We performed quality filtering for both single cell RNA-seq samples by excluding cell types with fewer than 5 cells per type. After filtering, we ended up with 17 subtypes for PDAC-A and 11 subtypes for PDAC-B. In the simulation experiment, we merged subtypes in PDAC-A to match with the names in PDAC-B sample. Specifically, ‘mDCs A’ and ‘mDCs B’ were merged as ‘mDCs’, as PDAC-B only has the ‘mDCs’ cell annotation. Similarly, we merged ‘Marcophages A’ and ‘Macrophages B’ as ‘Macrophages’, and ‘Cancer A’ and ‘Cancer B’ as ‘Cancer’. We ended up with 14 cell types, where ‘Acinar cells’, ‘T cells & NK cells’, ‘pDCs’, ‘Ductal - APOL1 high/hypoxic’ are missing in PDAC-B single-cell RNA-seq data and ‘Endocrine cells’ is missing in PDAC-A cells.We further categorized them into three region-level cell types with cancer cells, cuctal cells including all ductal subtypes, and other normal cells for all other nine cell types.

***Running deconvolution methods***

We ran each deconvolution method listed below in two modes: region level and finer cell type mode. In the region-level deconvolution mode, we used three references: internal, external, and ReSort. To ensure consistency and enable fair benchmarking, we used the same parameters wherever applicable for each tool when using different references.

The internal reference refers to the single-cell RNA-sequencing data (PDAC-A) used to generate the simulated ST data, which does not exist in reality. The external reference refers to the single-cell RNA-sequencing data from the same tissue but different samples (PDAC-B). The external reference represents the scenarios for most deconvolution tasks using the state-of-the-art methods listed below. The MIST reference is extracted from the ST data itself to avoid the technical effects caused by an external reference. The returned proportions of all cell types were further normalized to guarantee they were summed to one. All code needed to reproduce our results is available at GitHub (https://github.com/LiuzLab/ReSort_manuscript).

***Running RCTD***

We ran RCTD using the commands instructed by the authors at <https://github.com/dmcable/spacexr>. Specifically, we used the function creat.RCTD() with ten cores, and the function run.RCTD() with parameter “doublet mode = full” to enable estimating for more than two cell types.

***Running MuSiC***

We ran MuSiC by following the instructions (<https://xuranw.github.io/MuSiC/articles/MuSiC.html>) provided by the authors of the package. We used the music_prop() function with default parameters.

***Running stereoscope***

We followed the authors’ instructions at <https://github.com/almaan/stereoscope>. We used default values provided by the instructions except for reducing the epoch values to reduce the computational time and avoid overfitting. Instead, we used an epoch value of 10,000 for each experiment in training the reference and the ST data, while the default value is 75,000.

***Running SPOTlight***

We followed instructions to run the function spotlight_deconvolution(). Marker genes for every cell type were detected using the function FindAllMarkers() from the R package Seurat^32^. We normalized the raw count data by the library size and then transformed it into the log scale. Markers for each cell type were then extracted for genes with over 20% fold change and adjusted p-value of less than 0.05.

***Running SpatialDWLS***

We used the function runSpatialDeconv() from the R package Giotto. Using the same procedure to extract marker genes when running SPOTlight, we generated a signature matrix by first detecting marker genes for each cell type. We used default values for all other parameters.

***Running cell2location***

We followed the tutorial from https://cell2location.readthedocs.io/ to perform cell2location with our data. With the loading and basic preprocessing of the Visium and reference data, we fitted and extracted the cell type-specific expression signatures from the reference data using the RegressionModel() function with 800 epochs to ensure convergence. We then used the cell2location() function to estimate the abundance of cell types at each spot after training the cell2location model with 30,000 epochs. All the training was done in one GPU node and took 40-50 minutes.

***Running BayesSpace***

We ran BayesSpace by following its tutorial at <https://edward130603.github.io/BayesSpace/articles/BayesSpace.html>.We constructed a SingleCellExperiment object using raw counts and spatial coordinates. We used default parameters with log-normalized gene expression data, the top 2,000 highly variable genes, and seven principal components. Next, we extracted spot-level clusters using the function spatialCluster() with 10,000 iterations, and subspot-level clusters using function spatialEnhance() with 100,000 iterations.

***Running CIBERSORTx***

We ran CIBERSORTx^6^ with the library-size normalized count matrix without log-scale transformation, as suggested by Jing et al^15^. To run CIBERSORTx, we used the default LM22 reference data, with 1000 permutations.

***Evaluation of simulation methods***

We used Pearson’s correlation coefficient, referred to as ρ, to estimate the concordance between the estimated cell types’ proportions and the ground truth (Equation 6).

$$\rho= \frac{\sum\left( \mathbf{P}_{s}- \bar{\mathbf{P}} \right)(\mathbf{Y}_{s}- \bar{\mathbf{Y}})}{\sqrt{\sum\left( \mathbf{P}_{s}-\bar{\mathbf{P}} \right)^{2}\left( \mathbf{Y}_{s}- \bar{\mathbf{Y}} \right)^{2}}} (6)$$

$\mathbf{P}_{s}$ denotes the ground truth spot *s*′s proportion and $\mathbf{Y}_{s}$ represents the estimated spot *s*′s proportion. The metric *ρ* is used to assess the estimated values’ accuracy at the given spot *s*. We used the function stats.pearsonr() from the Python package scipy to calculate ρ.

The second metric we evaluated is the Kullback–Leibler (KL) divergence, also referred to as relative entropy and $D_{\mathrm{KL}}$ (Equation 7).

$$D_{\mathrm{KL}}= \sum_{t} \mathbf{Y}_{t}*\log\frac{\mathbf{Y}_{t}}{\mathbf{P}_{t}} (7)$$

$D_{\mathrm{KL}}$ captures the divergence between the ground truth distribution and the estimated distribution of cell type compositions.

A good method should have a high ρ score and a low $D_{\mathrm{KL}}$ score.

Moreover, to assess the models’ performance in detecting immune infiltration in the tumor region, we calculated the precision, recall, and F1 scores for two classes: infiltrated and pure tumor spots. An infiltrated tumor spot is a spot in the tumor region with a immune proportion of no less than 5%. A pure tumor spot is a spot in the tumor region with a tumor proportion greater than 95%. With the definition of these two classes, we calculated the precision, recall, and F1 scores for each class (Equation 8-10).

$$Precision= \frac{\#True Positive}{\#True Positive+\#False Positive} (8)$$

$$\mathrm{Recall}=\frac{\#True Positive}{\#True Positive+\#False Negative} (9)$$

$$F1= \frac{2*Precision*Recall}{Precision+Recall} (10)$$

We systematically evaluated the classification accuracy by showing each class's precision, recall, and F1 scores (Supp. Fig. 8). Additionally, we calculated a weighted F1 score defined as $F1_{\mathrm{weighted}}= \alpha*F1_{\mathrm{pure}}+\left( 1-\alpha\right)*F1_{infilt.}$, where $\alpha=\frac{N_{\mathrm{pure}}}{N_{\mathrm{infilt}}} ,$with $N_{\mathrm{pure}}$ representing the number of pure cancer spots and $N_{\mathrm{infilt}}$ defining the number of immune-filtrated spots within the tumor region.

***Applying ReSort on Pancreatic Ductal Adenocarcinomas Spatial Transcriptomics (PDAC-ST)***

PDAC-ST data (PDAC-A ST1) was obtained from Gene Expression Omnibus under accession number GSE111672: [GSM3036911](https://www.ncbi.nlm.nih.gov/gds/?term=GSM3036911%5bAccession%5d). We used the filtered raw count matrix to perform the analysis. We ran MIST to extract the spatial regions and used marker genes RM4SF1, CRISP3, and PRSS1 to annotate cancer, ductal and other normal regions based on the original study^16^ (Supp. Fig. 4). Region-level deconvolution was performed using ReSort pseudo-internal reference with Stereoscope. Finer cell type deconvolution used PDAC-B external reference with ReSort strategy. Results were compared with only using PDAC-B external reference.

***EMT Spatial Transcriptomics experiments***

PyMT-N and PyMT-M cells were derived from the spontaneous tumor of the MMTV-PyMT mouse model to represent epithelial and mesenchymal types, respectively. PyMT-N and PyMT-M cells mixed at a 1:1 ratio (2.5x105 cells/each) were resuspended in Matrigel and injected into the #4 mammary fat pads of C57/B6 mice. On day 21 after injection, the tumors were resected at approximately 1 cm after the mice were euthanized and perfused with PBS. The tumors were then excised and embedded with OCT in an isopentane bath kept in dry ice. The blocks were then sectioned at 10 µm thick and placed on the designated regions of a 10x Genomics Visium Spatial Gene Expression slide. After H&E staining, each section was imaged using a bright color field by Cytation 5. The sections were then processed following 10X Genomics gene expression protocols until the libraries were constructed, which were sequenced by Novaseq 6000 with 150bp paired-end reads. About 300M reads were recorded for each sample.

We used the software SpaceRanger-1.3.0 to process the mouse breast cancer ST samples with both epithelial-type and mesenchymal type tumors on the samples. After running SpaceRanger, the filtered count matrix was used for further analysis.

***Region detection and annotation for the PyMT-M-N* mouse breast cancer sample**

We preprocess the sample with MIST^14^ using the function preprocess(species=”Mouse”, hvg_prop=0.9, n_pcs=10), which removed uninformative genes with low coverage and variance. We then called the function extract_regions(sigma=0.4, min_region=3, gap=0.02), which extracted at least 40% of all spots and assigned them to at least three major regions.

We molecularly annotate the PyMT-M-N mouse breast cancer sample that have both epithelial and mesenchymal clones using previously published epithelial- and mesenchymal-associated markers^33^. We used Krt18 and Fn1 as epithelial transcriptomics markers, and Zeb1 and Twist1 as mesenchymal markers to label the epithelial region and mesenchymal region. Supp. Fig. 5 shows that substantially higher read counts from the green and red regions indicate these two are likely to be tumor regions, where cells having higher densities and are more proliferated. Moreover, we observed higher expression values of Krt18 and Fn1 in the red region, while Zeb1 and Twist1 are enriched in the green region, suggesting that red region being epithelial tumor and green region as mesenchymal tumor (Supp. Fig. 5). We assigned blue region as a stroma region because it has a low total number of gene counts and is not enriched with either epithelial or mesenchymal markers.

***Cell type deconvolution for the PyMT-M-N* mouse breast cancer sample**

After detecting and annotating the regions for the PyMT-M-N mouse breast cancer sample, we employed RCTD to first deconvolute the region-level cell types, including Epithelial-tumor, Mesenchymal-tumor and stroma cells’ proportions across the tissue. Next, due to the lack of a proper single-cell RNA-seq reference data, we used the LM22 immune signature matrix as an external reference to infer the relative immune proportions at each spot within the sample. Next, we used ReSort’s two-step strategy (See Method - *Finer cell type deconvolution*) to normalize the immune cell types and obtain the final estimates of each immune cell type and cancer subtype proportions.

***External validation using epithelial and mesenchymal-like TCGA cancer tumors***

We used the function getTCGA(disease="BRCA", data.type="RNASeq", type="RPKM") from the TCGA2STAT R package to obtain breast cancer RNAseq data^34^. We then extracted triple-negative breast cancer (TNBC) tumors using the meta information^27^, resulting in 128 tumor samples. We normalized the RPKM by the library size and performed log-transformation.

Then, we extracted top 100 epithelial-enriched marker genes and the top 100 mesenchymal-enriched marker genes from the ST samples to perform clustering on the 128 TNBC samples. Based on the clustering results from the R package pheatmap (Supp. Fig. 7), we extracted 20 epithelial-like and 22 mesenchymal-like TNBC tumors. We ran CIBERSORTx in the absolute mode to decompose immune cell types’ abundances in these 44 TNBC E- and M-like tumors.

***Immunohistochemistry staining on frozen primary tumors***

Tumor-bearing mice were euthanized and perfused with 30mL PBS before the primary tumors were removed and embedded immediately in OCT in an isopentane bath pre-chilled to

-80℃ and maintained on dry ice. Tumor sections at 10µm thickness were fixed with 10% neutral buffered formalin in PBS and permeabilized with 0.2% Triton X-100 in PBS for 10min at room temperature and blocked with 10% normal donkey serum in PBS-GT (PBS with 0.1% Triton X-100 and 0.1% gelatin) for 1 hour at room temperature. Sections were then incubated with primary antibodies (chicken anti-GFP, 1:200, Novus Biologicals, NB100-1614; rabbit anti-RFP, 1:200, Rockland, 600-401-379; goat anti-CD206, 1:100, R&D Systems, AF2535) overnight in a humidified chamber at 4℃. Slides were then washed and incubated with Alexa Fluor 488-conjugated donkey anti-chicken (1:400, Jackson ImmunoResearch), Alexa Fluor 555-conjugated donkey anti-rabbit (1:400, Jackson ImmunoResearch) and Alexa Fluor 647-conjugated donkey anti-goat (1:200, Jackson ImmunoResearch) secondary antibodies for 2 hours at room temperature in a humidified chamber. Sections were then washed and stained with Hoechst 33342 (Thermo Fisher Scientific 62249) and mounted with Prolonged Gold Antifade Mountant (Molecular Probe). Images were acquired by a Zeiss LSM780 confocal microscope.

**Supplementary Figures**
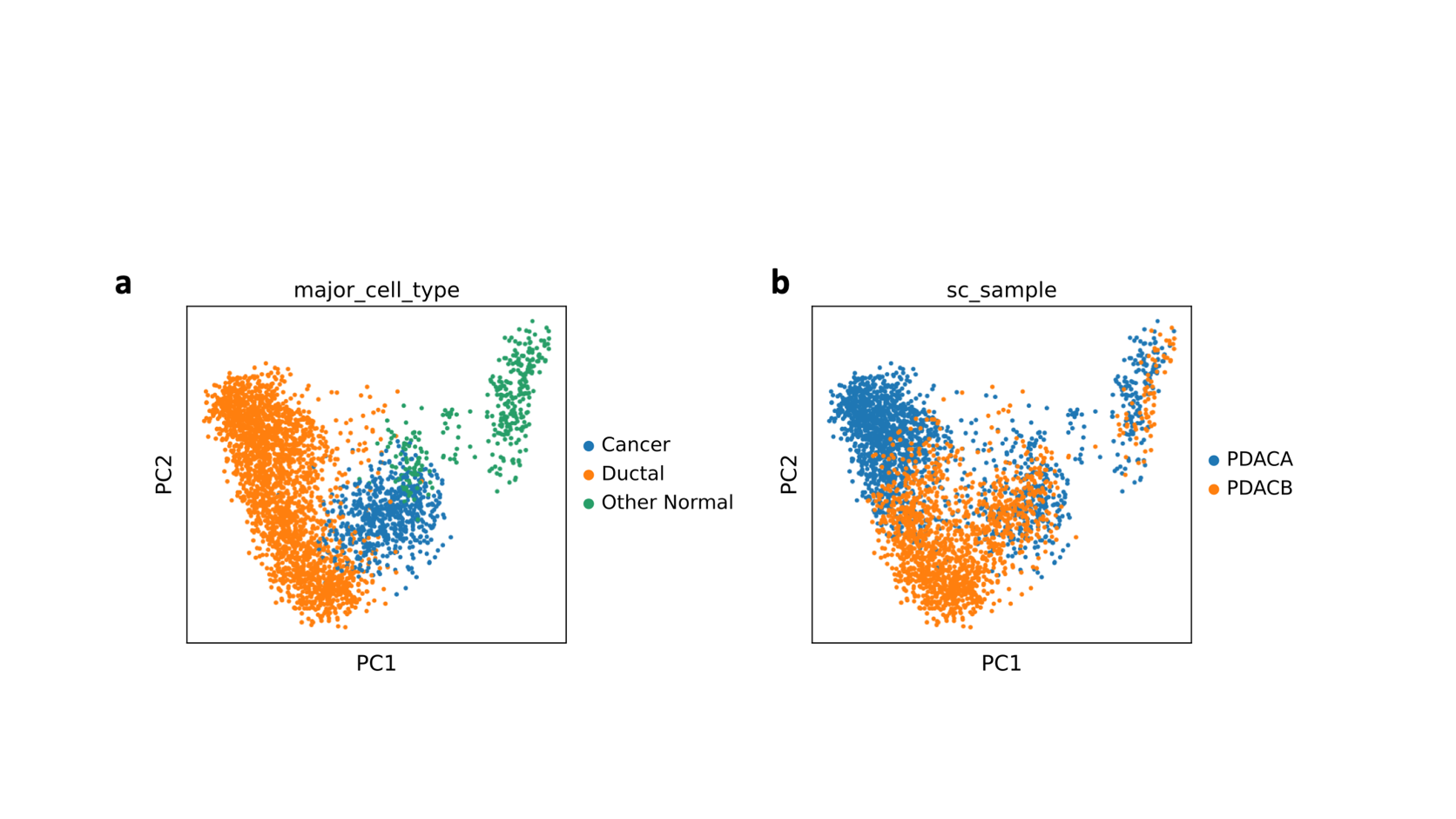


**Supplementary Figure 1 PCA plots of the pancreatic ductal adenocarcinomas**. **a**: PCA plot colored by region-level cell types. **b**: PCA plot colored by the single-cell RNA-seq sample IDs. Because of batch and platform differences, single-cell RNA-sequencing of these two samples showed significant heterogeneity between individuals.


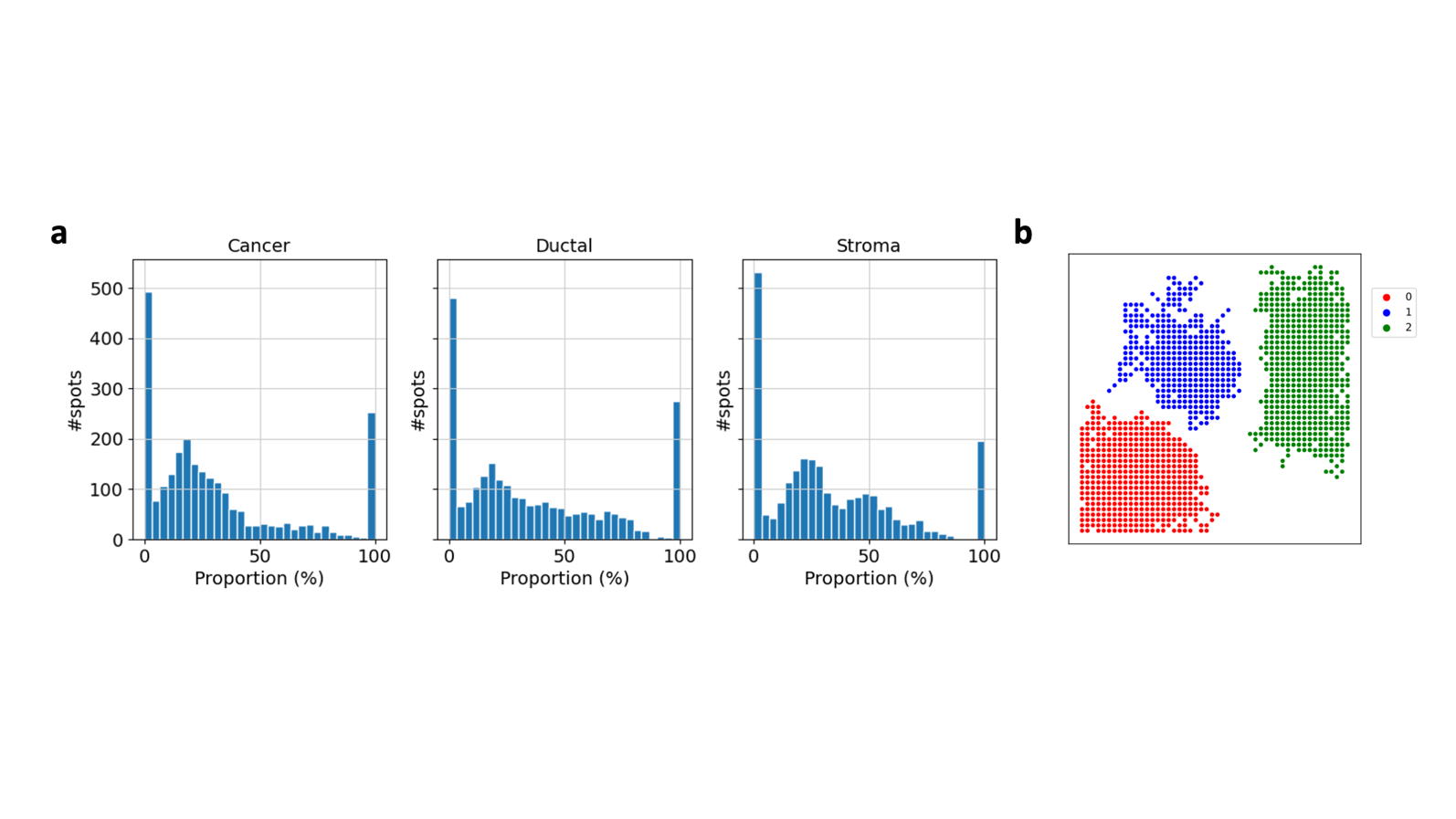


**Supplementary Figure 2 Simulation of ST samples using primary cell types**. **a**: Simulated proportional distributions of three primary cell types. **b**: MIST detected regional spots for three major cell types. Red: cancer; green: ductal; blue: other normal cells.


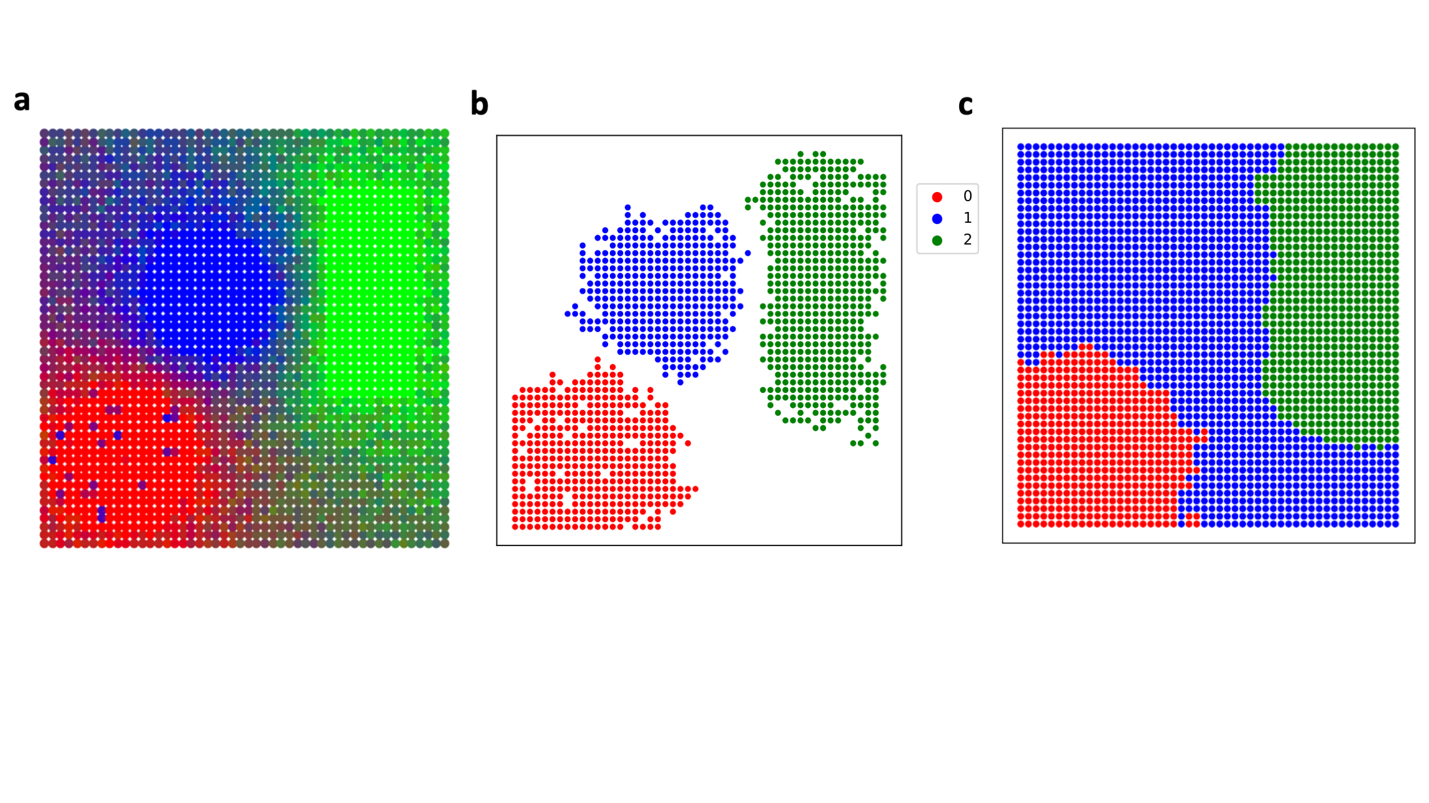


**Supplementary Figure 3** **Simulation of ST samples with immune cells infiltrating tumor spots.** **a** Spatial pattern of the simulated ST sample. Each spot is colored by region-level cell types’ proportions denoted by red as cancer, green as ductal and blue as other normal (including immune) cell types. **b** MIST detected regional spots for three major cell types. Colors are matched with (a). **c**: BayesSpace’s spatial clusters.


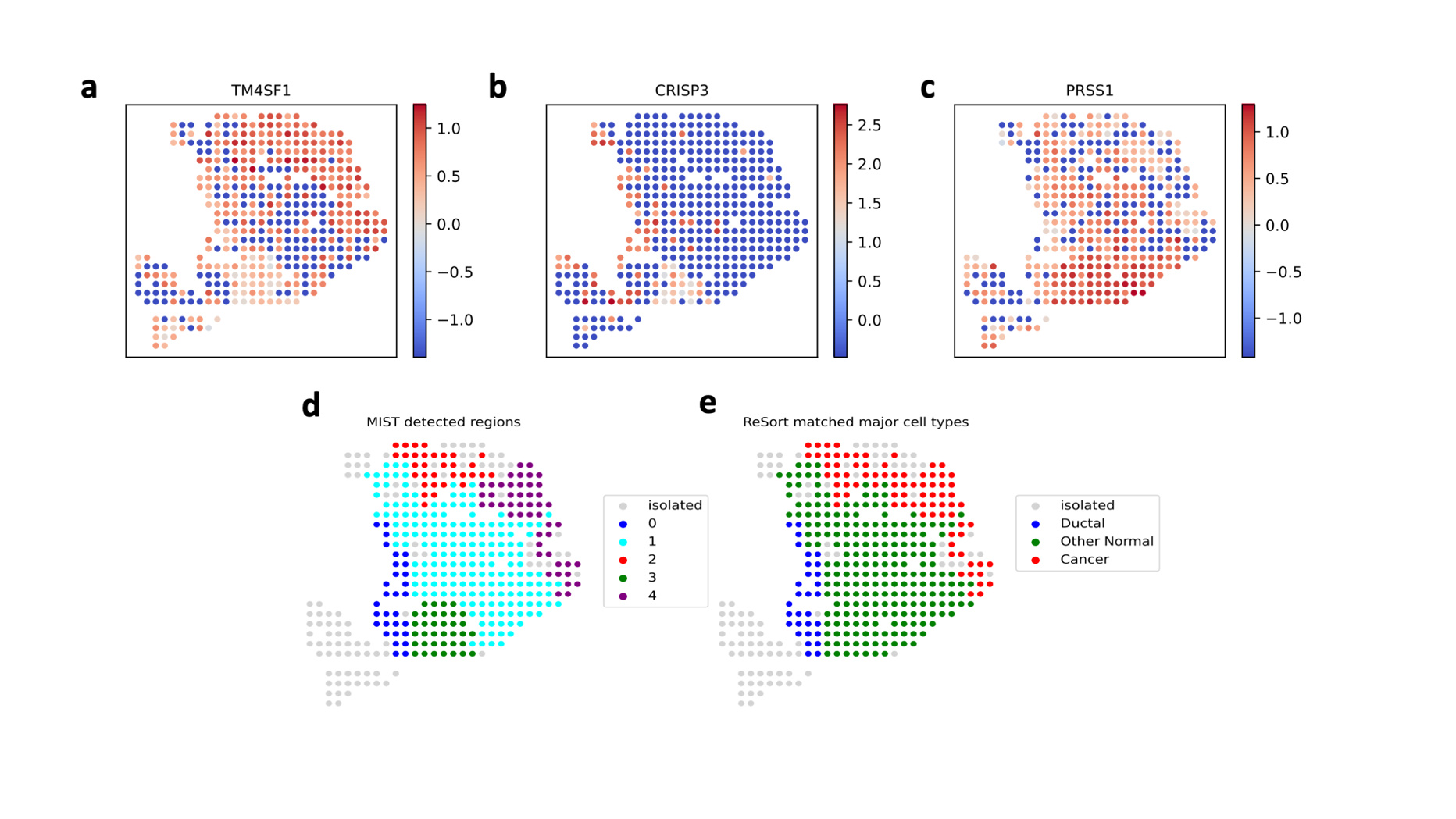


**Supplementary Figure 4 Region detection and annotation for the PDAC-A ST sample.**

**a – c** Marker genes’ expression patterns for cancer (TM4SF1), ductal (CRISP3) and other normal / pancreatic cells (PRSS1). **d** MIST detected regions. **e** Annotated regions using the three marker genes.


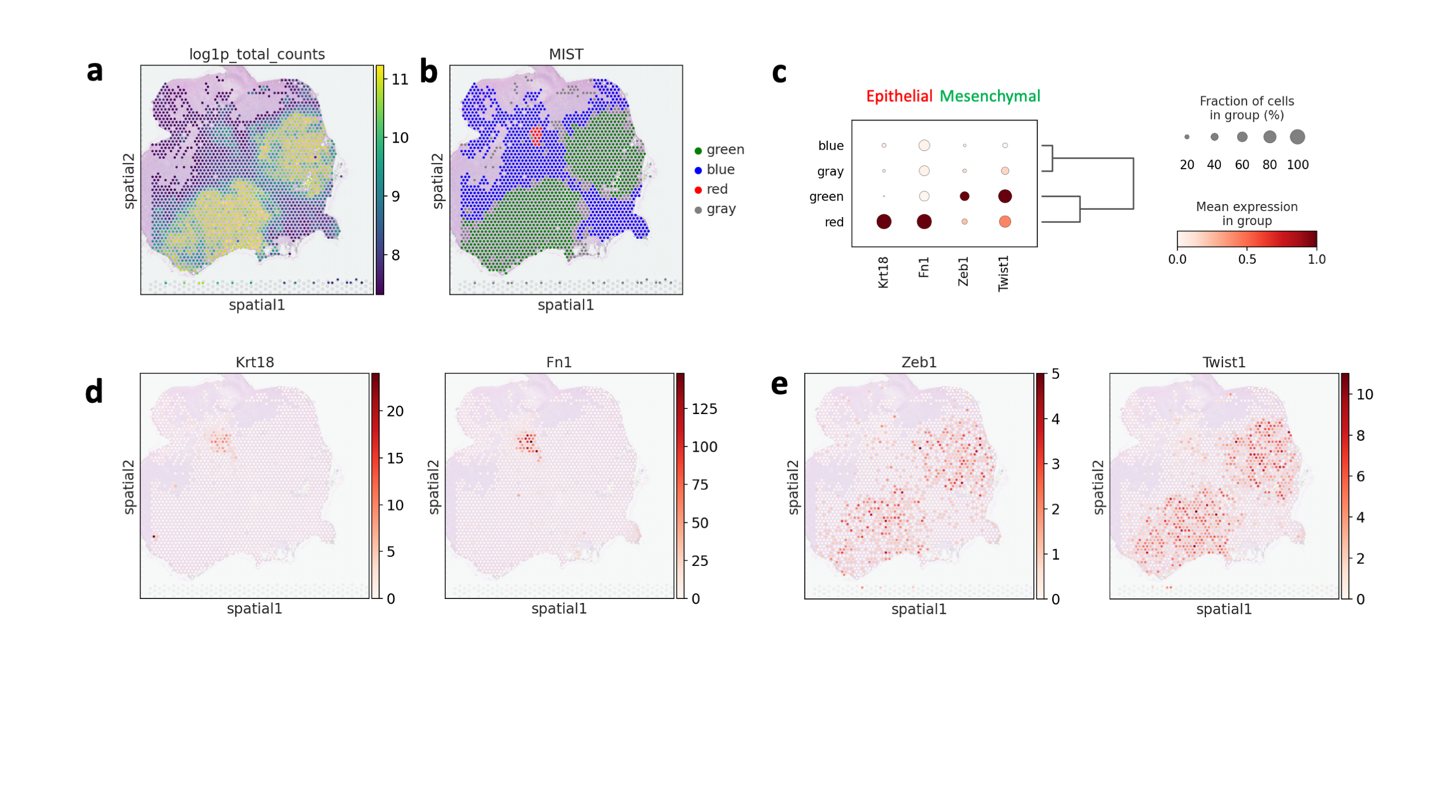


**Supplementary Figure 5** **Annotating PyMT-M-N breast cancer tumor. a** Heatmap of log-scaled total number of gene counts in the sample. **b** regions detected by MIST colored in green, blue and red colors. Gray colored spots are not assigned to any major regions. **c** Expression dot plot of epithelial (Krt18 and Fn1) and mesenchymal markers (Zeb1 and Twsit1). **d** Heatmap of epithelial marker genes’ (Krt18 and Fn1 expression with higher values colored in red. **e** Heatmap of mesenchymal marker genes’ (Krt18 and Fn1 expression with higher values colored in red.


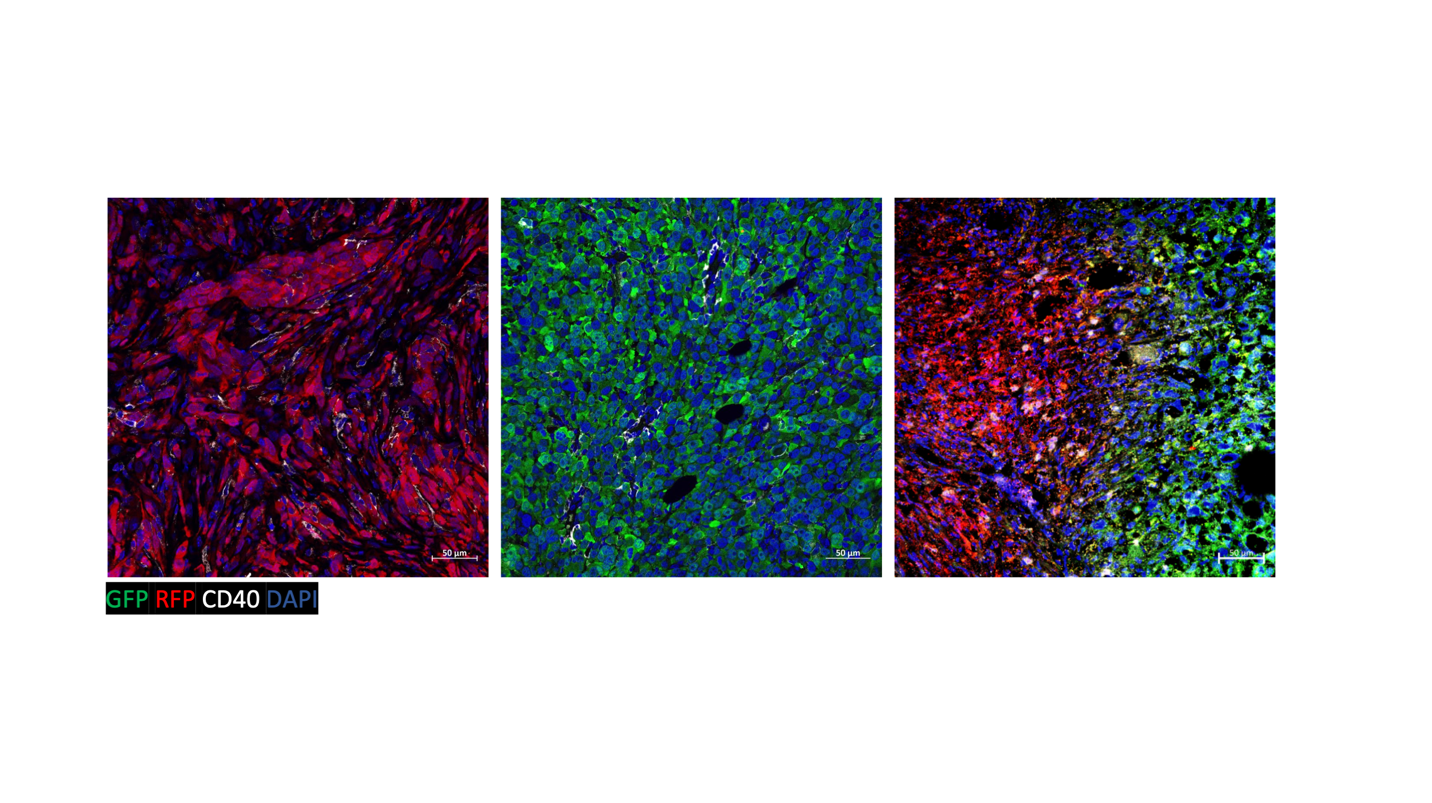


**Supplementary Figure 6** **Histological staining of M1 using CD40 in breast cancer tumor with epithelial and mesenchymal clones.** RFP: Epithelial; GFP: Mesenchymal; CD40: M1; DAPI: cell nucleus.


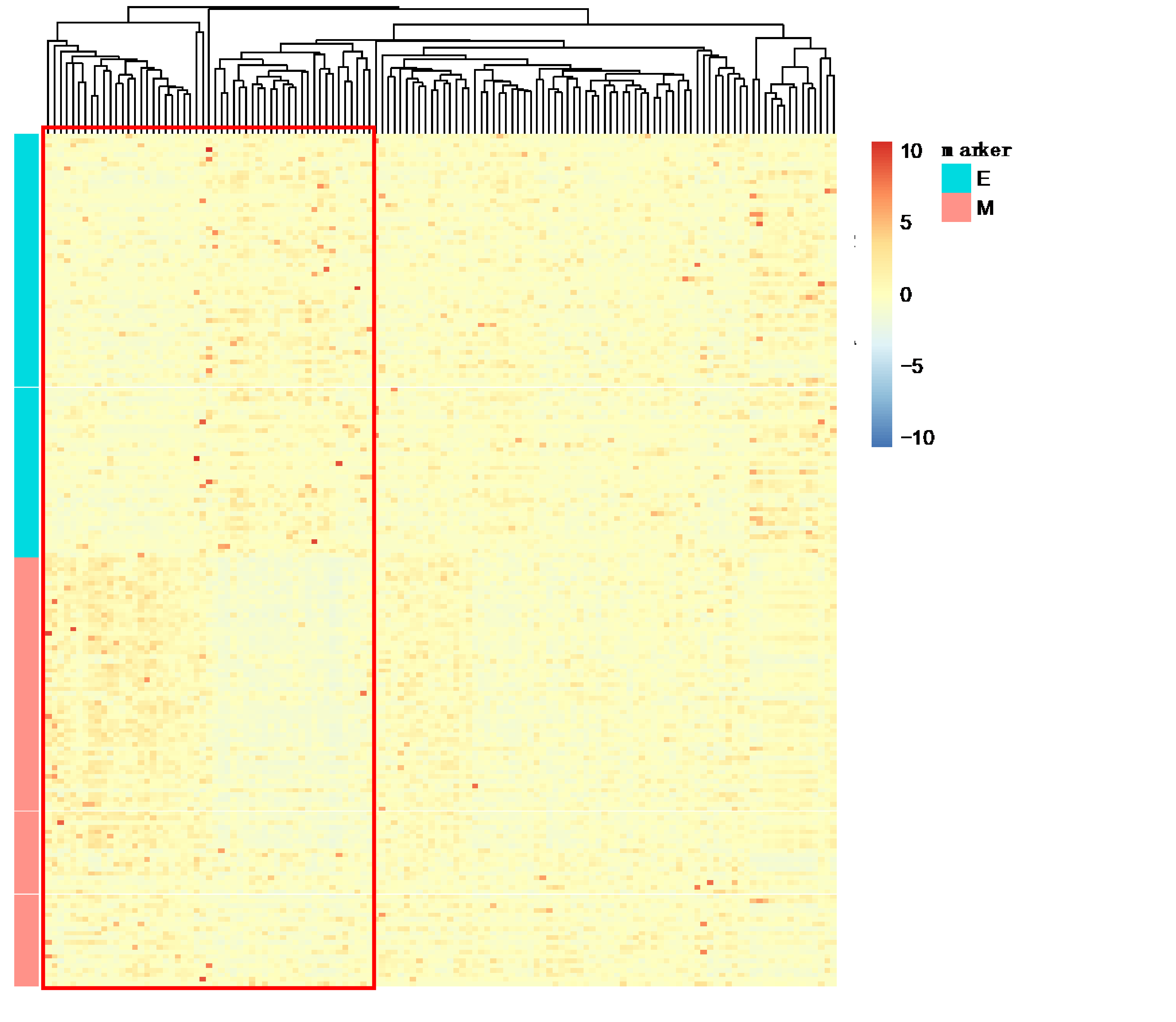


**Supplementary Figure 7** **Heatmap of epithelial and mesenchymal marker genes’ expression in 128 triple negative breast cancer (TNBC) tumors from TCGA.** Genes are annotated by marker groups in the row annotations. Columns of the red box are the 44 epithelial- and mesenchymal-like TNBC tumors used for further study.

**Supplementary Figure 8 Classification report on pure tumor and immune-infiltrating tumor spots.**
